# The burden of diabetes on the soft tissue seal surrounding the dental implants

**DOI:** 10.3389/fphys.2023.1136973

**Published:** 2023-02-16

**Authors:** Zhanwei Zhang, Chonghao Ji, Daobin Wang, Maoshan Wang, Dawei Song, Xin Xu, Dongjiao Zhang

**Affiliations:** ^1^ Department of Implantology, School and Hospital of Stomatology, Cheeloo College of Medicine, Shandong Key Laboratory of Oral Tissue Regeneration & Shandong Engineering Laboratory for Dental Materials and Oral Tissue Regeneration, Shandong Provincial Clinical Research Center for Oral, Shandong University , Jinan, China; ^2^ Taian Municipal Hospital, Tai’an, China; ^3^ School of Stomatology, Shandong First Medical University, Shandong Academy of Medical Sciences, Jinan, China

**Keywords:** diabetes, dental implants, soft tissue sealing, mucous integration, peri-implantitis, extracellular matrix

## Abstract

Soft tissue seal around implant prostheses is considered the primary barrier against adverse external stimuli and is a critical factor in maintaining dental implants’ stability. Soft tissue seal is formed mainly by the adhesion of epithelial tissue and fibrous connective tissue to the transmembrane portion of the implant. Type 2 diabetes mellitus (T2DM) is one of the risk factors for peri-implant inflammation, and peri-implant disease may be triggered by dysfunction of the soft tissue barrier around dental implants. This is increasingly considered a promising target for disease treatment and management. However, many studies have demonstrated that pathogenic bacterial infestation, gingival immune inflammation, overactive matrix metalloproteinases (MMPs), impaired wound healing processes and excessive oxidative stress may trigger poor peri-implant soft tissue sealing, which may be more severe in the T2DM state. This article reviews the structure of peri-implant soft tissue seal, peri-implant disease and treatment, and moderating mechanisms of impaired soft tissue seal around implants due to T2DM to inform the development of treatment strategies for dental implants in patients with dental defects.

## 1 Introduction

Diabetes mellitus is a group of metabolic diseases characterized by hyperglycemia. Chronic high blood glucose levels are associated with physical damage and failure of various organs and tissues and are one of the leading causes of death worldwide (Awad et al., 2021). Data released by the International Diabetes Federation in 2021 show that the world population with diabetes is approximately 463 million (20–79 years old) (Schacter and Leslie, 2021). Diabetes seriously affects patients’ quality of life and life expectancy and has become a severe public health problem (Zheng et al., 2018a; Hu and Jia, 2018). The vast majority of diabetes cases can be divided into two major classes, type 1 diabetes mellitus (T1DM) and type 2 diabetes mellitus (T2DM) (American Diabetes Association, 2013). T1DM is an autoimmune chronic disease, and its cause is an absolute deficiency of insulin due to the autoimmune destruction of the pancreatic beta cells (Rodrigues Oliveira et al., 2023). In distinction, T2DM is an endocrine metabolic disorder, accounting for approximately 90%–95% of all diabetic patients (American Diabetes Association, 2019), due to resistance to insulin action and inadequate compensatory insulin secretory response (American Diabetes Association, 2013).

Implant therapy is gradually becoming the restorative option for patients with missing or edentulous teeth for its advantages, such as comfort and aesthetics. The longevity and long-term functional stability of implant dentures depend on the integration of the implant with the bone and the soft tissue barrier that protects the implant and the alveolar bone from external stimuli. The soft tissue is firmly attached to the implant, promoting its aesthetic and functional stability, and is an essential factor in the long-term outcome of the implants (Zhao et al., 2014). In recent years, peri-implant soft tissue seal has received increasing attention. However, relevant clinical and basic research is scarce, and this unique implant-mucosa interface’s role and formation process are still unclear.

Nevertheless, complications inevitably occur during implant restoration and later denture function, including surgical, mechanical, and biological complications (Romanos et al., 2019). Biological complications of implant prostheses refer to inflammatory damage to the soft and bone tissues surrounding the implant, mainly including peri-implant mucositis and peri-implantitis (Kwon et al., 2020). Peri-implantitis is an inflammation of the peri-implant mucosa and progressive peri-implant bone loss initiated by pathogenic bacteria and is the leading cause of implant detachment (Buser et al., 2017). The oral mucosa protects the periodontal tissues against bacteria and other harmful substances. Studies have revealed that peri-implant soft tissue inflammation can induce pathological changes at the implant-surrounding tissue interface, causing resorption of the peri-implant bone (Chackartchi et al., 2019). In short, a suitable implant denture must perform the occlusal function and form an excellent soft tissue barrier to achieve the aesthetic requirements and produce a protective effect.

The literature on diabetes and obesity reports that up to 50% of dental implants may be affected by peri-implantitis, that diabetes rises the risk of peri-implantitis, and that poor glycemic control significantly elevates the rate of implant failure (Moy et al., 2005; Ferreira et al., 2006; Daubert et al., 2015; Monje et al., 2017). Patients with glucose levels greater than 8.0% HbA1c at the time of implant placement have a detrimental effect on bone metabolism and implant integration rates, with a 2-fold delay in implant integration rates (Aguilar-Salvatierra et al., 2016). Although implants are initially stable in diabetic and obese patients, implant-bone integration is reduced, making the implant more susceptible to peri-implantitis (Chrcanovic et al., 2014; Coelho et al., 2018). In addition to this, the diabetic state may increase the pathogenic flora and exacerbate the local inflammatory response, increasing the susceptibility and severity of peri-implant mucosal tissue and bone, and inflammation extending from the soft tissue margins may exacerbate the alteration of the implant-bone interface, further accelerating the bone loss associated with peri-implantitis (Nibali et al., 2022). And especially, differing from T1DM, which did not adversely affect the incidence of inflammation (Sannino et al., 2020), marginal bone loss and implant survival in dental implants, patients with T2DM had increased clinical and radiological peri-implant indices such as plaque index (PI), bleeding on probing (BOP), probing depth (PD) and marginal bone loss (MBL) after implant surgery compared to the non-diabetic group (Alrabiah et al., 2018). As a result, T2DM patients may be at higher risk of implant loss associated with peri-implantitis.

A detailed dissection of the physiological structure and microstructure of the implant-gingival interface is presented here, presenting evidence for the influence of pathogenic bacteria, gingival inflammation, and epithelial migration on soft tissue seal, and the effect of diabetes on peri-implant soft tissue sealing is analyzed in detail. It is essential to note that some molecular mechanisms are explicitly mentioned in this review, implying that this could be a potential target for ameliorating soft tissue seals.

## 2 The importance of the soft tissue seal in dental implant therapy and peri-implant disease

Since Professor Branmark introduced the concept of osseointegration 50 years ago (Brånemark et al., 1969), dental implants have evolved into a successful and predictable treatment modality for replacing missing teeth (Buser et al., 2017). The key to achieving good clinical results with restorative implant treatment is establishing tight osseointegration, such as direct structural and functional contact between the surface of the load-bearing implant and the ordered arrangement of bone tissue. Dental implants preserve the adjacent tooth structure and bone and can improve chewing function and quality of life in edentulous and partially edentulous patients with restorations (Jivraj and Chee, 2006; Battle-Siatita et al., 2009; Jofre et al., 2013; Hartlev et al., 2014). As a result, oral implant restoration has become an increasingly popular option for restoring missing teeth in patients with missing teeth or edentulous jaws (Tarnow, 2014; Buser et al., 2017).

A unique feature of implants is a mucosa-penetrating component, whereby the abutment forms a transition zone between the implant and the crown, penetrating the soft tissue. In addition to osseointegration, the soft tissue interface surrounding the implant plays a critical role in the long-term success of implant-supported restoration (Chackartchi et al., 2019). These soft tissues separate the peri-implant bone from the oral environment to avoid disruptions in the balance that could lead to the penetration of pathogenic bacteria causing peri-implant disease or bone loss.

The peri-implant disease usually presents in two forms: peri-implant mucositis and peri-implantitis. Peri-implant mucositis is an inflammatory reaction of the peri-implant mucosa without marginal bone loss around the implant; without proper care, it can progress to a more severe condition called peri-implantitis (Matarazzo et al., 2018). Moreover, peri-implantitis is a chronic inflammatory disease caused by dental plaque, a destructive host response caused by local, systemic and environmental factors, including soft tissue inflammation and progressive loss of supporting bone, which can promote osteoclast-mediated bone resorption and inhibit osteoblast-mediated bone formation, resulting in progressive loss of peri-implant bone and is the primary cause of implant loss (Buser et al., 2017). Peri-implant tissues exhibit a more pronounced inflammatory response than teeth with the same amount of plaque, and reversing this inflammation takes longer in the peri-implant tissues (Salvi et al., 2012; Ivanovski and Lee, 2018). Pathogenic bacterial infestation is the initiating factor of peri-implantitis and the leading cause of biological complications in implant dentures (Dreyer et al., 2018; Daubert and Weinstein, 2019).

Implant dentures have been developed as a tool for the dental rehabilitation of patients with diabetic edentulism. Good osseointegration and bio-containment of the soft tissues around the implant are essential for long-term success and stability. The soft tissue-implant integration is the primary defense of the implant against adverse external stimuli and is a fundamental biological basis for oral implants (Chackartchi et al., 2019).

## 3 The structure of the soft tissue seal around dental implants

### 3.1 The soft tissue seal in the gingiva–abutment interface

When implants are placed, the healing process occurs, such as connective tissue repair, while epithelial cells move horizontally to cover the wound on the connective tissue (Atsuta et al., 2005a; Sculean et al., 2014; Tomasi et al., 2014). Since the implant blocks the growth and horizontal migration of epithelial and connective tissue, they begin to spread along the implant surface at depth and turn back at a certain depth, thus forming a mucosal tissue similar to that of natural teeth, i.e., the pores formed by the implant are gradually sealed by epithelial and connective tissue (Fujii et al., 1998; Gulati et al., 2020).

Histologically, the peri-implant mucosa consists of well-keratinized oral epithelium, sulcular epithelium and a thin barrier epithelium facing the abutment, which corresponds to the around the tooth, called the peri-implant Junctional Epithelium (JE) (Schroeder et al., 1981; Gould et al., 1984; Buser and Brägger, 1989). The height of the peri-implant JE is approximately 2 mm, and the connective tissue below the JE is about 1.5 mm. Thus, the average biological width usually reaches 3.8 mm (Berglundh and Lindhe, 1996). When the biological width of any part of the peri-implant mucosa decreases, marginal bone resorption is generally observed, so the biological width is adjusted to compensate for these changes. The desmosome junctions in JE are fewer than the oral epithelium, so the intercellular gaps are wide and contain many immune cells such as neutrophils (Shimono et al., 2003). The JE is the first line of defense of the titanium or zirconium oxide peri-implant tissue against pathogen invasion. The epithelial tissue attaches to the surface of the implant system *via* the inner basement membrane and hemidesmosomes (HDs) (Ivanovski and Lee, 2018; Ren et al., 2019). The HDs are mainly located on the root side of the JE, so the implant’s JE is weakly closed (Bartold et al., 2000; Atsuta et al., 2013; Atsuta et al., 2016).

Among the connective tissue surrounding the implant, it is noteworthy that, due to the absence of the osseous layer, there are no vertical fibers inserted vertically into the implant surface, and the gingiva is directly “adapted” to the implant surface (Fujii et al., 1998), indicating that the sealing capacity of this structure is more fragile than that of a natural tooth and more prone to periodontal rupture and subsequent bacterial invasion (Moon et al., 1999). Compared to natural teeth, fewer fibroblasts and mesenchymal cells are usually intermediate to collagen fibers (Al Rezk et al., 2018). The connective tissue is adhered by gingival fibroblasts to the surface of the implant system through adhesive plaque attachment, tight junctions and extracellular matrix connections, and collagen fibers parallel to the implant surface (Bartold et al., 2000; Amberg et al., 2018).

### 3.2 The molecular structure of the soft tissue seal

#### 3.2.1 Epithelial tissue

The gingival epithelial cells in the JE rely mainly on the Internal Basal Lamina (IBL) and HDs structures to attach to the surface of the implant system (Bartold et al., 2000). The gingival epithelium secures the internal basal lamina and is divided into a dense and hyaline layer.

The main components of the dense layer are Laminin 332 (Laminin 5) and Collagen XVIII (BP180, BPAG2). Laminin 332 is an important molecule closely related to epithelial cell adhesion and migration and is involved in the attachment of peri-implant epithelium to the implant surface (Ikeda et al., 2000; LeBleu et al., 2007). Laminin is a family of macromolecular glycoproteins composed of disulfide-linked heterotrimeric chains (*α*, *β*, *γ*) that form a cross-like shape (Atsuta et al., 2005a; Aumailley et al., 2005; Atsuta et al., 2005b; Aumailley, 2013; Yamada and Sekiguchi, 2015; Abdallah et al., 2017). It acts as a ligand for Integrins, linked to integrin α6β4 *via* the α3 chain to form HDs and adhere to the extracellular matrix. The peptide coating containing Laminin 332 directly induces the sealing of keratinocytes around dental implants (Koidou et al., 2018). BP180 is a transmembrane protein adjacent to integrins and plays a vital role in maintaining the link between intracellular and extracellular structural elements involved in adhesion.

Lamina Lucinda is part of the basal lamina and consists of five major components: partial integrin α6β4, partial BP180, plectin, CD151, and BP230 (BPAG1e) (Ghohestani et al., 2001). Integrin α6β4 is a non-covalent heterodimer that is a transmembrane component of HDs (Davis et al., 2001; Holmes and Rout, 2011). The extracellular region α6 of integrin binds to BP180, CD151 and laminin 332 (Rezniczek et al., 1998; Kazarov et al., 2002). Integrin β4 mediates intracellular interactions between plakin family members plectin and BP230 and the keratin cytoskeleton attached to the transmembrane protein BP180. HDs are highly specialized integrin-mediated epithelial attachment structures that allow cells to adhere firmly to the implant surface and establish connections between the epithelial keratin cytoskeleton and the hyaline layer below (Fontao et al., 2001; Koster et al., 2004).

In the intracellular part of the HDs, part of the plectin and BP230 form the dense external block, which, together with the keratin filament-linked subunits and keratin filaments, are called the thick internal block. HDs become the transmembrane connection between the tooth or implant and the gingiva. JE forms a protective barrier for the mechanical stability of the tooth or dental implant and a physical barrier against biofilm invasion (Walko et al., 2015; Atsuta et al., 2016). However, they are distributed almost exclusively in the lower part of the JE at the implant interface, so the peri-implant epithelial seal is significantly weaker than the periodontal tissue. Gingival epithelial cells upregulate the expression of adherent plaques and HDs at early time points, thus forming an infection-free seal surrounding dental implants (Atsuta et al., 2005a; Pendegrass et al., 2015).

#### 3.2.2 Connective tissue

Between the apical edge of the JE and the bone tissue, there is a 200 µm wide zone of connective tissue divided into a central and a lateral zone. And the 40 µm wide central site immediately adjacent to the implant surface contains a lower volume of collagen and a higher density of elongated fibroblasts but no blood vessels (Buser et al., 1992). The connective tissue under the JE contains collagen I, III, IV, and VII. Collagen I is the main component of the apical connective tissue of the peri-implant mucosa, and collagen V is more abundant in the peri-implant tissue (Chavrier and Couble, 1999). The collagen fibers of the connective tissue are parallel to the long axis of the implant, thus creating only a physical adaptation without any biological integration/insertion and having less adhesion (Chavrier et al., 1994). In addition, the limited number of gingival fibroblasts around the implant may prolong the healing, regeneration and maturation cycle of the connective tissue, allowing bacterial invasion (Al Rezk et al., 2018). As the main cellular component of the connective tissue layer surrounding the implant, gingival fibroblasts are responsible for the secretion and remodeling of the extracellular matrix (ECM), especially collagen fibers. After surgery, gingival fibroblasts begin proliferating, repopulating, and producing collagen-rich ECM in the submucosal region attached to the implant surface (Romanos et al., 1995). Collagen fibril formation continues for approximately 4–6 weeks, followed by forming a mature connective tissue seal between 6 and 12 weeks (Fujii et al., 1998; Al Rezk et al., 2018). In conclusion, the low number of gingival fibroblasts results in poor integration of the transmucosal connective tissue layer on the implanted dental abutment due to the lack of fibrous connections to the abutment surface.

Gingival fibroblasts adhere to the implant surface mainly through adhesive plaque attachment, tight junctions and ECM attachment. The adhesion patch complex consists of proteins communicating with the ECM, the transmembrane protein integrins, and the intracellular cytoskeleton (Burridge et al., 1988). The seven signs, represented by talin, paxillin, and vinculin, regulate gene expression to stabilize the adhesion complex through a feedback system and transmit different signals from the ECM to the cytosol (Hynes, 2002; Owen et al., 2005). To obtain enhanced connective tissue integration, upregulation of fibroblast activity and enhanced expression of integrins α5, β1, and β3 are required to ensure the early establishment of direct fibrous connections.

**FIGURE 1:**
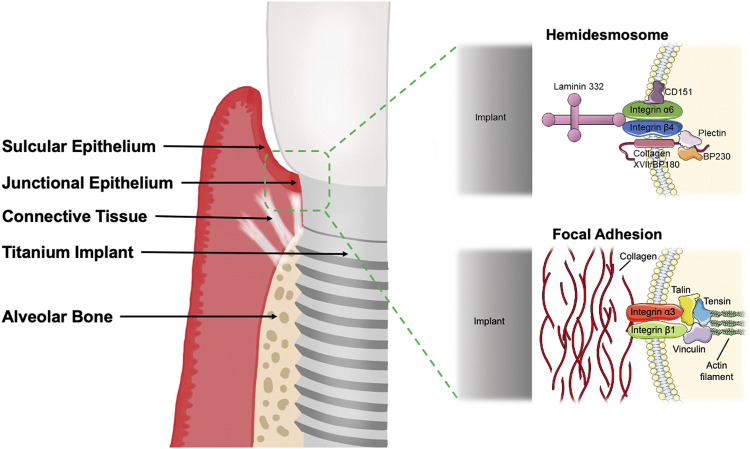
The soft tissue seal around the implant consists of gingival junctional epithelium and connective tissue. The peri-implant junctional epithelium adheres to the implant mainly through hemidesmosome structures, while the gingival fibroblasts exert adhesion mainly through focal adhesions. The figure was partly generated using Servier Medical Art, provided by Servier, licensed under a Creative Commons Attribution 3.0 unported license. BP180, Bullous pemphigoid antigen 180; BP230, Bullous pemphigoid antigen 230.

The firm attachment of soft tissues to the implant (as shown in Figure 1), which ultimately allows complete closure of the subgingival bone tissue and protection from the oral environment, promotes implant stability and is the most critical factor influencing the long-term maintenance of dental implants (An et al., 2012). Studies are scarce to quantify the strength of cell-implant adhesion and evaluate the role of attachment proteins. In conclusion, soft tissue closure as a biological barrier is vital in remodeling soft and hard tissues around implants and the long-term maintenance of clinical implants.

## 4 The mechanisms of diabetes leading to impaired soft tissue seal

### 4.1 Subgingival microbiome and bacterial susceptibility affected by diabetes

Studies have found an increase in *Actinomyces naeslundi* and *Streptococcus oralis* around implants in healthy patients compared to natural teeth (Gürlek et al., 2017). There are still inconsistent and even contradictory conclusions about whether diabetes alters the bacterial composition of the oral cavity (Chapple et al., 2013; Graves et al., 2020). In type 2 diabetic periodontitis, the detection rate of orange complex species in subgingival plaque was significantly higher compared to a healthy periodontal state (Sabancı et al., 2022). A 16s rRNA assay of the subgingival flora of patients with periodontitis showed a significant augment in the relative abundance of *Aggregatibacter*, *Neisseria*, *Fusobacterium,* and *Actinomycetes* in type 2 diabetic patients (Casarin et al., 2013). Another report indicated that the levels of *Porphyromonas gingivalis* (*P. gingivalis*) or *Porphyromonas forsythia* (*P. forsythia*) showed an increase (da Cruz et al., 2008). Furthermore, a recent metagenomic shotgun sequencing analysis comparing the subgingival flora of non-diabetic and diabetic patients found that subgingival flora tended to be more abundant in pathogenic species in the T2DM state, regardless of whether the periodontal tissue was healthy, suggesting that T2DM patients face a higher risk of developing periodontitis (Shi et al., 2020).

Similarly, diabetes may also affect peri-implant inflammation by altering the subgingival flora. One study divided patients suffering from peri-implantitis into a systemic health group and a T2DM group (Sabancı et al., 2022). There are no significant differences in microbial species were found in the shallow peri-implant pockets of the two groups (Sabancı et al., 2022). However, *Campylobacter rectus* (*C. rectus*), *P. gingivalis*, *Actinobacillus actinomycetemcomitans* (*A. actinomycetemcomitans*) and *Tannerella forsythia* (*T. forsythia*) may be reduced in deep pockets around implants by T2DM (Sabancı et al., 2022). An interesting study to identify the microbiota in peri-implantitis pockets by matrix-assisted laser desorption/ionization time-of-flight mass spectrometry showed *Neisseria flavescens*, *Streptococcus constellatus*, *Slackia exigua*, *Streptococcus intermedius*, *Fusobacterium nucleatum,* and *Gemella morbillorum* were the central resident folk detected in the peri-implantitis pockets (Yeh et al., 2019). The above findings suggest that the sequencing identification of bacteria alone may not determine whether they are pathogenic in patients with type 2 diabetes. Non-etheless, the diabetic state produces changes in some microbial species in the peri-implant pocket, and these changes may affect microbial homeostasis, but the exact effects are inconclusive.

Since the discovery of bacterial binding to ECM proteins (e.g., collagen, fibronectin, and laminin) more than 40 years ago (Kuusela, 1978), our understanding of pathogen-host cell interactions has gradually enhanced. The adhesion of bacteria to host tissues is the first decisive step in the infection process (Keller et al., 2015). In Gram-negative bacteria, adhesion to host cells can be achieved by binding to ECM proteins as a tool for pathogen-host contact (Vaca et al., 2020). Even pathogens secrete bacterial proteases to degrade ECM proteins and thus disrupt the barrier (Singh et al., 2012). For example, *Treponema denticola* produces chymotrypsin-like proteases targeted to degrade Laminin, collagen IV, and fibrinogen, allowing bacteria to invade the basement membrane after ECM degradation (Bamford et al., 2007; Singh et al., 2012). In the oral cavity, JE can express defensins, chemokines and cytokines (Groeger and Meyle, 2019). When bacteria colonize JE, the adhesion of keratinocytes is negatively affected, and laminin and collagen present in JE become binding sites for pathogens (Marre et al., 2020). In conclusion, the difference between hyperglycemia and normoglycemia is mainly in the deep pockets around the implants (Sabancı et al., 2022), and diabetic patients are more susceptible to microbiome changes than normal healthy individuals (Sabancı et al., 2022).

### 4.2 Increased immuno-inflammatory response in diabetes

Diabetes increases the physical inflammatory response (Andriankaja et al., 2012; Pacios et al., 2012). Diabetic patients have increased tumor necrosis factor-α (TNF-α), elevated polymorphonuclear leukocyte infiltration, and more bone loss in diabetic rats than in normoglycemic rats (Kang et al., 2012). Injection of equal amounts of bacteria into the soft tissues of diabetic animals caused a more severe inflammatory response than in normal animals (Naguib et al., 2004). These results suggest that diabetic animals have a more dramatic host response to bacterial attacks.

Diabetes augments levels of inflammatory cytokines such as TNF-α, interleukin-1β (IL-1β), interleukin-17 (IL-17), interleukin-23 (IL-23), and interleukin-6 (IL-6) in human periodontal tissue (Bastos et al., 2012; Polak and Shapira, 2018). Increased resistance and permeability of the gingival epithelial cell layer after TNF-α and IL-1β treatment is associated with the development of gingival inflammation (Fujita et al., 2012; Miyagawa et al., 2016; Lagha and Grenier, 2019). Moreover, human gingival fibroblasts (HGF) treated with TNF-α and IL-1β induced increased HGF infiltration (Lv et al., 2020). Enhanced expression of inflammatory cytokines leads to rose vascular permeability and inflammatory cell recruitment (Domingueti et al., 2016) with upregulated receptor activator of nuclear factor kappa-B ligand (RANKL) or decreased osteoprotegerin (OPG) expression, stimulating increased bone resorption (Bastos et al., 2012). The more severe the hyperglycemia, the higher the levels of IL-1β and IL-6 in the oral saliva and the greater the probability of peri-implantitis (Al-Askar et al., 2018; Al-Sowygh et al., 2018; Vissink et al., 2018). And the restraint in anti-inflammatory factors may be one of the reasons for the exacerbation of periodontitis. Regulatory T-cells and M2-type macrophages responsible for anti-inflammation produce anti-inflammatory factors such as interleukin-4 (IL-4), interleukin-10 (IL-10), transforming growth factor-β (TGF-β) and anti-inflammatory lipid mediators, significantly reduced in diabetic complications (Acharya et al., 2017; Van Dyke, 2017).

Diabetes also affects the innate and adaptive immune responses of cells. Neutrophils make up the majority of cells in the gingival sulcus and are an essential component of the host response to tooth-associated biofilms (Hajishengallis, 2014). High glucose stimulates the production of more chemokines, which induce neutrophil recruitment in response to bacterial challenges (Cintra et al., 2014; Manosudprasit et al., 2017; Zheng et al., 2018b), and stimulates neutrophil initiation by increasing protein kinase C (PKC) activity (Karima et al., 2005). In addition, diabetes augments neutrophil activation and reactive oxygen species (ROS) production to increase damage to periodontal tissue (Guerra and Otton, 2011). Macrophages are associated with peri-implant disease. Diabetes promotes the production of IL-1β and TNF-α by macrophages, which may contribute to the enhancement of peri-implant infection (Salvi et al., 1998). Diabetes may increase the polarization of M1 macrophages to upregulate the susceptibility and severity of peri-implantitis (Sreedhar et al., 2017). In addition, higher levels of TNF-α, CC chemokine receptor 5 (CCR5) and CXC chemokine receptor 3 (CXCR3) at peri-implant sites in patients with chronic periodontitis and diabetes suggest a high potential for peri-implant bone loss (Venza et al., 2010). Dendritic cells are also associated with peri-implant disease, and their function may be regulated by diabetes, thereby promoting the disease process. Diabetes may control dendritic cells to alter alveolar bone loss by increasing the production of Th1 or Th17 lymphocytes or decreasing the formation of regulatory T-cells (Santos et al., 2010; Silva et al., 2012; Song et al., 2018).

A fundamental cause of the pathogenesis of peri-implantitis is how diabetes affects the soft tissue barrier of the implant, but it has not received sufficient attention. The sealing structure formed by the soft tissues is an effective barrier to protect the implant from external microorganisms, but the corresponding specific mechanisms remain to be thoroughly investigated.

### 4.3 High-level matrix metalloproteinases in diabetes

Matrix Metalloproteinases (MMPs) are a group of protein hydrolases that play an essential role in the turnover of ECM and maintain the balance between remodeling and degradation of ECM (Klein and Bischoff, 2011; Bastos et al., 2017). The substrate of MMP is mainly collagen, but it also includes many other ECM proteins, including fibronectin, laminin, etc. (Ruoslahti, 1996; Stamenkovic, 2003). Regarding the mechanism of neurological damage by cerebral ischemia, MMP-9 destroys neurons by degrading laminin (Lee et al., 2009). MMPs are associated with diabetes-related peri-implantitis. MMP-8 and MMP-9 levels in oral fluids can reflect the disease status of periodontal disease to some extent (Domenyuk et al., 2019). In particular, the level of MMP-8 in oral fluid is upregulated proportionally to the severity of periodontal/peri-implant disease (Sorsa et al., 2000; Kiili et al., 2002; Sorsa et al., 2010; Sexton et al., 2011; Sorsa et al., 2011). MMP-8 in saliva was similar in composition to its counterpart within the gingival sulcus (Gangbar et al., 1990), and a similar pattern of elevated MMP-8 was observed in the gingival sulcus of peri-implantitis to that observed at the site of periodontitis (Ma et al., 2000; Arakawa et al., 2012; Janska et al., 2016). A study reported that patients with both chronic periodontitis and diabetes had much higher levels of MMP-8 and MMP-9 in periodontal tissue compared to patients with chronic periodontitis but systemically healthy patients and healthy controls (Kumar et al., 2006), and there was a trend toward significantly increased MMP-8 in patients transitioning from the absence of both diseases (Hardy et al., 2012). In addition, the excessive release of various MMPs by fibroblasts affected by reactive oxygen species promoted the degradation of connective tissue and bone matrix (Guan et al., 2009; Kaur et al., 2013). The interaction of MMPs with advanced glycosylation end-products (AGEs)/receptors for advanced glycosylation end-products (RAGEs) further contributes to increased inflammation in diabetes. AGEs accumulate and destroy periodontal tissue continuously under hyperglycemic conditions and directly or indirectly mediate intracellular effects through RAGE receptors of epithelial cells, gingival fibroblasts and other cells (Plemmenos and Piperi, 2022). The combination of AGEs and RAGEs in specific cell lines can stimulate the production and activity of MMPs, making the inflammatory response more severe (Ryan et al., 1999; Nesto and Rutter, 2002). AGEs can also influence the production and structure of ECM proteins by affecting the cross-linking of collagen (Snedeker and Gautieri, 2014).

### 4.4 Wound healing delayed by diabetes

Both oral mucosal and skin wound healing requires four stages: Hemostasis, inflammation, proliferation, and maturation/stromal remodeling. Soft tissue healing after implant placement surgery takes 6–8 weeks, which is much longer than the epithelial healing time for dental surgical wounds (7–14 days) (Salvi et al., 2000; Hämmerle et al., 2014). Wound healing involves re-epithelialization, massive new connective tissue formation and new bone formation (Ko et al., 2021). The diabetic oral wound healing process is characterized by impaired keratinocyte proliferation and migration, altered levels of inflammation, and reduced neo-connective tissue and bone formation (Ko et al., 2021). The factors involved in delayed wound healing in diabetes include hypoxia, fibroblast and epidermal cell dysfunction, impaired angiogenesis, and neovascularization, elevated MMPs, damage from ROS and advanced glycosylation end-products (AGEs), neuropathy and decreased multilevel host immune resistance (Lan et al., 2008; Chrcanovic et al., 2014; Brizeno et al., 2016). Most of these conditions are exacerbated in diabetes mellitus with poor glycemic control and significantly increase the risk of poor wound healing in dental procedures. An *in vitro* study confirmed that hyperglycemia significantly inhibits the adhesion and proliferation of human gingival fibroblasts on titanium (Liu et al., 2021). Diabetic wounds have more neutrophils, enhanced TNF expression levels, and declined expression of growth factors in wound fluid, which may delay wound re-epithelialization (Siqueira et al., 2010; Romanos et al., 2019). In cutaneous wounds, diabetes reduced the switch from the M1 macrophage phenotype to the M2 macrophage phenotype (Al-Mulla et al., 2011). More pro-inflammatory macrophages implied increased expression of inflammatory cytokines (e.g., IL-1, IL-6, and IL-8) and MMP, which may lead to more prolonged inflammation (Wilkinson et al., 2019). Genes involved in apoptosis, mucosal migration and intercellular communication were reported to be upregulated 4-fold–200-fold when inflammation around the implant increased, which simultaneously slowed the rate of wound healing (Ganesan et al., 2022).

### 4.5 Excess reactive oxygen species in diabetes

Chronic exposure to high glucose induces cytokine expression and responses to cytokine stimuli in diabetic patients, including the production of ROS (Lim et al., 2017; Zheng et al., 2018b). ROS are a series of molecular oxygen derivatives with pleiotropic properties, classified into radical ROS (e.g., hydroxyl radicals, superoxide anion radicals O_2_^−^, peroxyl radicals, and alkoxyl radicals) and non-radical ROS (e.g., H_2_O_2_, organic hydroperoxides, single linear state molecular oxygen, ozone, hypochlorous acid, and hypobromous acid) (Forrester et al., 2018; Hawkins and Davies, 2019; Lushchak and Lushchak, 2021). Physiological levels of ROS regulate various life processes in cells and organs, including cell proliferation, differentiation, migration, and angiogenesis (Niki, 2016). However, when ROS levels exceed physiological concentrations, it will lead to cell growth arrest and death (Niki, 2016; Li et al., 2021). Due to high intracellular glucose levels, the mitochondrial electron transport chain is overactive, inducing the formation of reactive oxygen species and producing and releasing inflammatory factors. Reactive oxygen species can induce apoptosis and damage cellular and stromal DNA and structural components (Yang et al., 2015). Increased mitochondrial reactive oxygen species production exacerbates periodontitis in diabetic patients (Sun et al., 2017).

### 4.6 Elevated advanced glycation end products in diabetes

**FIGURE 2:**
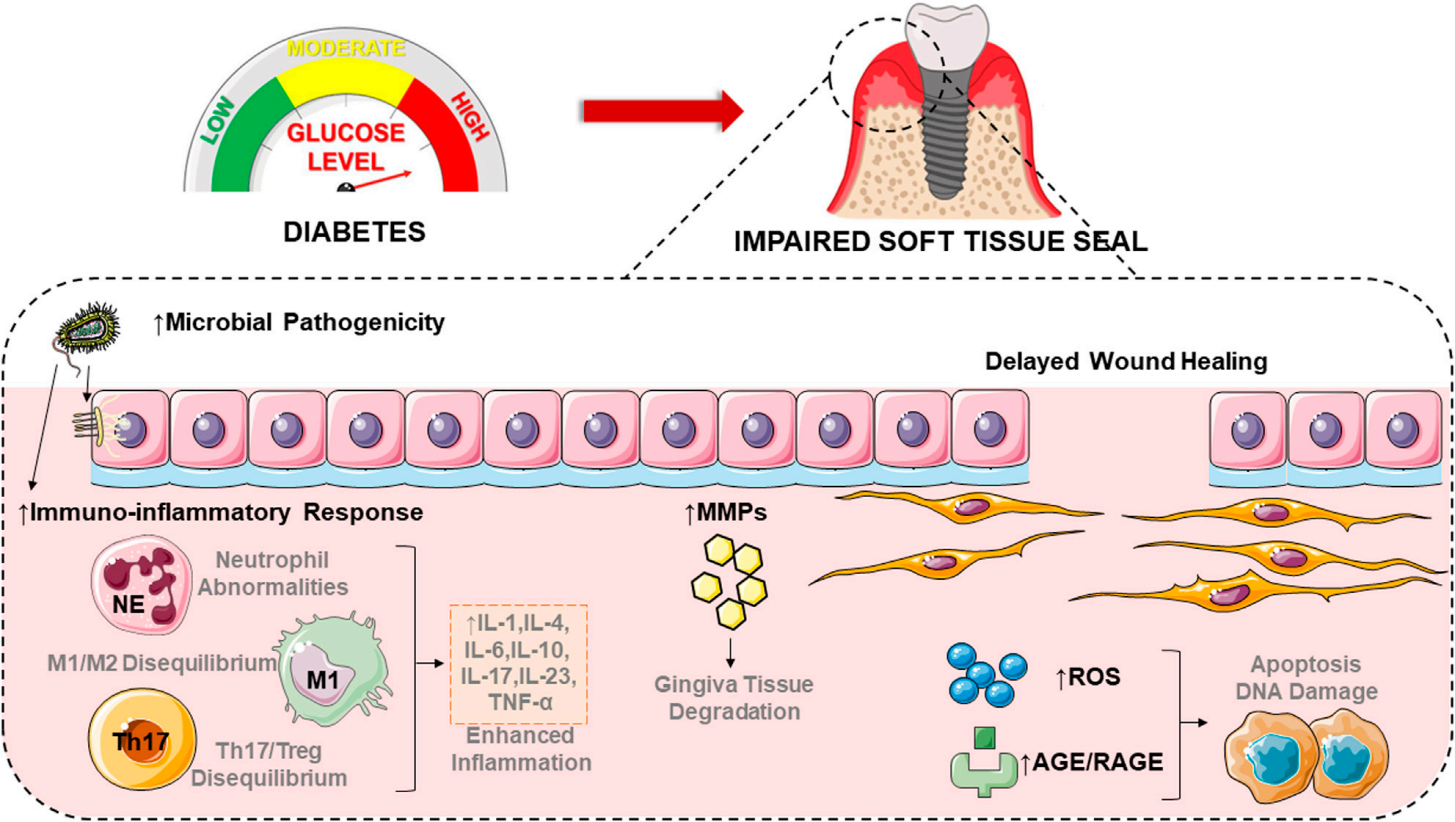
Diabetes affects the soft tissues around implants through a variety of mechanisms, including altering bacterial colony composition, promoting inflammatory responses, elevating matrix metalloproteinase concentrations, delaying the rate of wound healing, and increasing oxidative stress. The figure was partly generated using Servier Medical Art, provided by Servier, licensed under a Creative Commons Attribution 3.0 unported license. IL-1, interleukin-1; IL-4, interleukin-4; IL-6, interleukin-6; IL-10, interleukin-10; IL-17, interleukin-17; IL-23, interleukin-23; TNF-α, tumor necrosis factor-α; MMPs, matrix metalloproteinases; ROS, reactive oxygen species; AGE, advanced glycosylation end-product; RAGE, advanced glycosylation end product.

High glucose levels can lead to the formation of advanced glycosylation end products (AGEs). AGEs accumulate in most tissues of diabetic patients, including kidney, retina, gums, bone and periodontal tissue (Schmidt et al., 1996; Lalla and Papapanou, 2011; Napoli et al., 2017). AGEs bind to receptors for advanced glycosylation end products (RAGEs) and other receptors, activate nuclear factor κB (NF-κB), stimulate the production of reactive oxygen species, and induce the expression of inflammatory cytokines such as IL-6 and TNF-α (Singh et al., 2014; Nonaka et al., 2018). AGEs may inhibit the viability of human gingival fibroblasts and reduce the expression of type I and type III collagen (Ren et al., 2009). Interestingly, one study observed an increased concentration of AGEs in the fluid around the implant in the diabetic group and a significant correlation with the depth of the pocket around the implant (Al-Sowygh et al., 2018). In osteoblasts, excess AGEs promote apoptosis and inhibit osteogenic activity (Alikhani et al., 2007). Inhibition of RAGE reduced TNF-α production, which confirms the role of RAGE receptors in slowing the development of periodontitis (Lalla et al., 2003). A report on periodontitis and systemic disease found that combining AGEs and RAGEs leads to excessive inflammatory response and periodontal tissue destruction in patients with type 2 diabetes (Chapple et al., 2013). The effects of diabetes on dental implants’ soft tissue sealing are shown as follows (Figure 2).

## 5 Clinical implications and future perspectives

The soft tissue around the transmural part of the dental implant is remodeled so that the bone tissue around the implant is separated from the oral cavity, creating a biological width that serves as a defense mechanism against bacteria, such as soft tissue seals. This closure prevents inflammatory diseases around the implant and ensures a healthy condition and stable osseointegration. The damaging effect of diabetes, one of the risk factors for peri-implantitis, on peri-implant soft tissue seal may be reflected in the altered composition of the subgingival microbiome, a more intense host inflammatory response, impaired wound healing processes, and an excess of reactive oxygen species (ROS) and advanced glycosylation end products (AGEs). More importantly, excessive inflammatory factors and matrix metalloproteinases (MMPs) production promote the ablation of the extracellular matrix (ECM), which to some extent hinders the adhesion of epithelial and fibrous tissues to the implant surface achieved based on ECM structures such as hemidesmosomes and focal adhesion. However, there are no targeted methods to resist the development of peri-implantitis in diabetic patients, especially by preserving the ECM structures. Therefore, remodeling the adhesion structures of the peri-implant soft tissues is seen as a more effective potential direction. Understanding the effects of diabetes on gingival epithelial cells and gingival fibroblasts and thoroughly exploring the mechanisms involved are essential for developing and improving therapeutic approaches, which are suitable for establishing and maintaining soft tissue seals for the prevention of peri-implantitis and the longevity of successfully osseointegrated implants in diabetic patients. Reduced strength and time delays in soft tissue integration can affect the long-term stability of implant dentures. Researchers have never stopped searching for ways to improve the success of dental implants in diabetic patients, especially by focusing their studies on resisting the development and progression of diabetic peri-implantitis, and focusing on the improvement of soft tissue sealing is necessary to improve outcomes.
